# Clinical Validation of Non-invasive Simulation-Based Determination of Vascular Impedance, Wave Intensity, and Hydraulic Work in Patients Undergoing Transcatheter Aortic Valve Replacement

**DOI:** 10.1007/s10439-024-03635-5

**Published:** 2024-11-19

**Authors:** Jonathan Y. Brown, Gabriela Veiga Fernandez, Jose M. De La Torre Hernández, Michael Murphy, Benjamin S. Wessler, Elazer R. Edelman

**Affiliations:** 1 Institute for Medical Engineering and Science, Massachusetts Institute of Technology, 77 Massachusetts Ave Building E25-RM442, Cambridge, MA USA; 2 Clinical and Translation Science Institute, Tufts University, Boston, MA USA; 3 Tufts Medical Center, Boston, MA USA; 4 Division of Cardiovascular Medicine, Department of Medicine, Brigham and Women’s Hospital, Boston, MA USA; 5 Harvard Medical School, Boston, MA USA; 6 Cardiology Division, Hospital Universitario Marques de Valdecilla, IDIVAL, Santander, Spain

**Keywords:** Aortic input impedance, Vascular afterload, Aortic stenosis, Simulation, Non-invasive

## Abstract

**Purpose:**

The impact of Aortic Stenosis (AS) on the left ventricle (LV) extends beyond the influence of the pressure drop across the stenotic valve, but also includes the additional serial afterload imposed by the vascular system. Aortic input impedance is the gold standard for comprehensively studying the contribution of the vascular system to total myocardial afterload, but in the past measurement has been challenging arising from the need for invasive catheterization or specialized equipment to precisely record time-resolved blood pressure and flow signals. The goal of this work was to develop and validate a novel simulation-based method for determining aortic input impedance using only clinically available echocardiographic data and a simple blood pressure measurement.

**Methods:**

A simulation-based method to determine vascular impedance was developed using echocardiographic data and a brachial blood pressure measurement. Simulation-based impedance was compared to impedance calculated from echocardiographic flow data and pressure data from a non-invasive central pressure measurement device.

**Results:**

In validation analysis comparing patient-specific simulation-based vascular impedance to non-invasively measured impedance, correlation between methods across a range of vascular parameters varied between *R*^2^ = 0.40 and 0.99. A tendency was seen toward underestimation of pressure waveforms in point-by-point comparison of measured and simulated waveforms with an overall mean difference of 4.01 mmHg.

**Conclusions:**

Requiring only non-invasive clinical data that are widely available, simulation-based vascular impedance has the potential to allow for easier, more widespread, and larger-scale investigation of the effect of vascular impedance on total LV afterload.

**Supplementary Information:**

The online version contains supplementary material available at 10.1007/s10439-024-03635-5.

## Introduction

Aortic Stenosis (AS) is the most comment type of valvular disease in the western world [26]. Furthermore, worldwide there was a 433% increase in the prevalence of calcific aortic valve disease between 1990 and 2019 [17]. The advent of new technologies such as Transcatheter Aortic Valve Replacement (TAVR) for minimally invasive valve replacement and the observation that many patients have poor outcomes after aortic valve replacement (AVR) have prompted the search for enhanced diagnostics to identify disease early but also to determine potential benefit from intervention [1]. Pressure drop across the valve is not sufficient to predict need for benefit from TAVR, and there is evidence that vascular afterload adds further physiologic and prognostic information to understanding AS [6, 11, 24, 27]. It is though not clear that blood pressure alone can provide insight in this domain as 50% of patients post-TAVR are hypertensive, and paradoxically post-TAVR hypertension confers a significant mortality benefit over those that are normotensive [16]. Taken together, these observations motivate the need for a greater understanding of left ventricle (LV) vascular coupling that may be a key component to ensuring good outcomes post-TAVR.

Aortic input impedance provides the most comprehensive physiologic description of the interaction between the LV and vascular system. While the concept of input impedance has been in use since the early 1950s, a significant hurdle to its wide spread implementation has been difficulty in measurement and calculation [20]. Traditional determination of input impedance requires simultaneous central aortic blood pressure and blood flow through the aorta. Each of these time-based signals is then decomposed into the frequency domain using signal processing techniques such as Fourier transformation [20]. For each decomposed harmonic, the ratio of pressure and flow amplitudes was taken, and phases were subtracted to obtain the full-frequency domain-based impedance spectrum. In the past, obtaining concurrent pressure and flow signals has required invasive catheterization which is not conducive to repeated measurements required for tracking disease progression. While methods have been developed using non-invasive central blood pressure devices and echocardiography [3, 15] or Cardiac Magnetic Resonance [10] for blood flow for less invasive assessment, they require specialized equipment and training. They are also an additional time burden on the already-overloaded clinical workflow, making routine use difficult. Furthermore, given the highly dynamic environment of the cardiovascular system and high-dimensional nature of impedance, the direct relationship between various impedance parameters and clinical outcomes remains unclear in AS.

To allow for more accurate tracking of changes in impedance during the progression of AS, prior to TAVR, and the relationship to clinical outcomes, we sought to develop and validate a patient-specific simulation-based method to measure aortic input impedance [6]. Routine standard of care clinical echocardiographic data along with a simple brachial blood pressure measurement were used as inputs. Reference measured impedance was calculated using data from a non-invasive central pressure measurement device and echocardiograms. Our novel simulation-based method was validated in a cohort of AS patients undergoing TAVR.

## Materials and Methods

Patients undergoing TAVR were recruited from two centers in Spain (HUMV, Santander) between 2019 and 2020. Inclusion criteria were a diagnosis of severe symptomatic AS and suitable femoral access as determined by the local heart team. The study was approved at each site, written and informed consent was obtained from all subjects. Patients not able to provide informed consent were excluded.

### Echocardiographic Data

Echocardiographic data were captured at baseline prior to the TAVR. Standard clinical echocardiographic views and metrics were captured describing LV, valvular, and hemodynamic states. Specific data extracted for use in the calculation of impedance included peak aortic valve velocity, acceleration time, heart rate, stroke volume, and aortic valve area (AVA). Stroke volume and AVA were calculated via standard continuity-based methods [2].

### Non-Invasive Central Pressure Measurements

Non-Invasive central pressure measurements were captured using the SphygmoCor XCEL (AtCor Medical Sydney, Australia) system alongside echocardiographic data. Time-resolved central pressure waveforms were exported and saved for offline analysis for comparison against output central blood pressure waveforms from the Simulation-based vascular impedance (SBVI) method. Additionally, the standard brachial blood pressure was taken and recorded for use in the SBVI.

### Vascular Impedance

Recorded vascular input impedance was calculated via the standard method [20]. Time-resolved blood pressure and linear blood velocity flow waveform data were decomposed computationally into their Fourier harmonics via the Fast Fourier Transform (FFT). Input impedance amplitude values were calculated as the ratio of the pressure waveform amplitude at each harmonic to the flow amplitude. The impedance phase angles were calculated by subtracting the pressure and flow harmonic phase angles. Central aortic pressure waveform data were supplied from the SphygmoCor device. Linear velocity blood flow waveforms were generated using data extracted from echocardiographic exams using a 5th-order B-spline (Online Appendix Fig. 1). Heart rate was used to define the signal period, peak aortic valve velocity, the peak of the flow waveform, and acceleration time, the time to peak flow. The ejection duration (ED) was determined by an optimization algorithm that iteratively tested a range of ED values until the area under the curve matched the stroke volume as recorded by echocardiography. Further details on blood flow waveform generation can be found in the Online Appendix.

### Simulation-Based Vascular Impedance

**Fig. 1:**
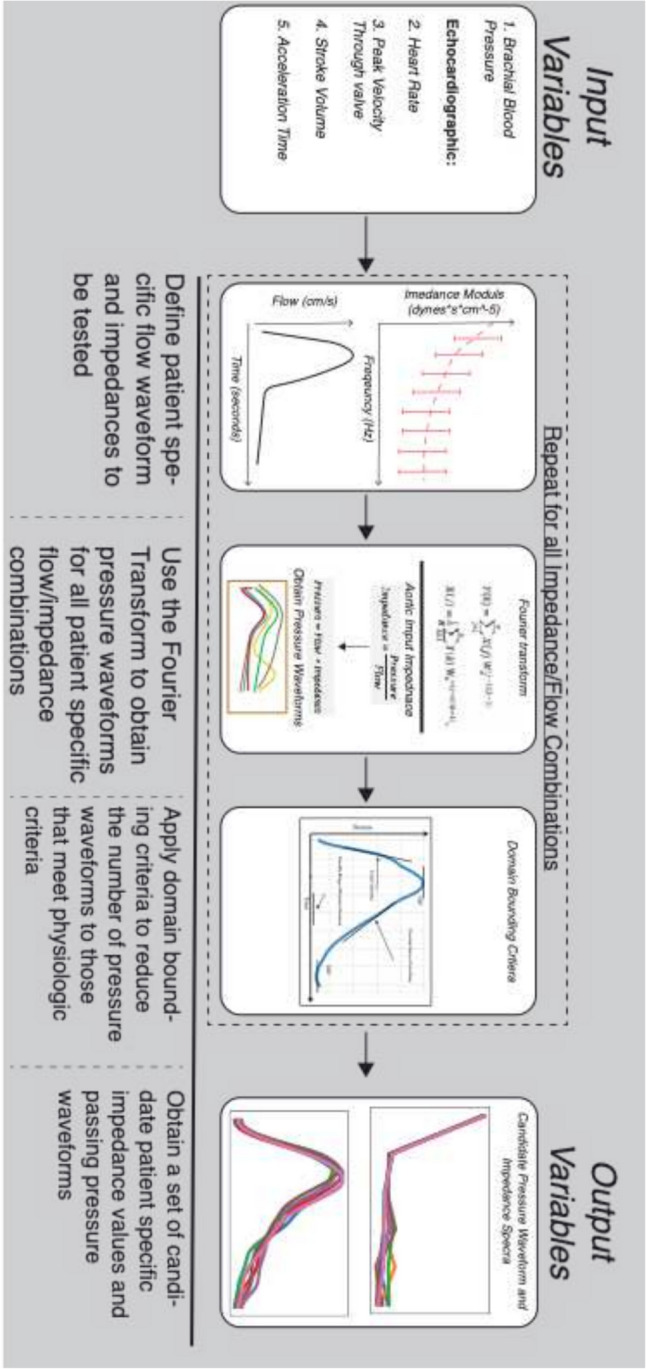
Impedance simulation workflow diagram

Simulation-based vascular impedance was calculated using an iterative method. Human vascular impedance spectrums, amplitudes, and phases fall within a set range. These ranges were defined based on values taken from literature both in normal [5, 20] and patients with AS [3, 6, 24]. With this knowledge, we created a set of candidate impedance spectrum amplitudes and phase values. The impedance spectrum range was then expanded by 20% to ensure all physiologic and pathophysiologic vascular states were represented. Five hundred million candidate amplitude and phase pairs were generated to ensure sufficient coverage across all possible combinations. Using the standard method of calculating impedance amplitude and phase using the FFT, we can rearrange each equation to solve for the pressure amplitude and phase harmonics. By applying the inverse FFT, we can obtain the time-resolved central pressure waveform. Each candidate set of impedance values was then used in this workflow along with the flow amplitude and phase calculated from the decomposed echocardiographic flow waveforms to obtain all-candidate central pressure signals. Using the well-validated generalized transfer function [13], this central pressure waveform was then converted into the corresponding brachial pressure waveform. Domain bounding criteria were applied to this signal to reduce the number of solutions to only those that were patient-specific and displayed a set of physiologic characteristics present in all human pressure waveforms (Fig. 1).

Domain bounding criteria were broken down into patient-specific and knowledge-based criteria. First candidate waveforms were filtered with the patient-specific criteria of the measured brachial blood pressure set to a tolerance of + /− 2.5 mmHg. Physiologic knowledge-based criteria required that (1) all pressure waveforms fall after the end of the systolic portion of the cardiac cycle, (2) peak pressure not occur after the end of systole, (3) the difference between initial pressure and minimum pressure be less than 1 mmHg, (4) the first local maximum after peak pressure if one exists be no greater than 3 mmHg above the first local minimum after peak pressure, (5) the first local pressure maximum be the global pressure maximum, and (6) the maximum pressure occur after the peak flow (Online Appendix Fig. 6). If multiple waveforms passed all criteria, median values were taken and used for analysis. All simulations were conducted using a custom Python code (Python 3.6).

### Vascular Parameters

A set of vascular parameters derived from each type of impedance calculation were used to allow for additional comparisons of SBVI and non-invasively recorded methods. Metrics selected for the comparison were derived both from the frequency domain representation of impedance and the time domain-based central pressure waveforms. Characteristic impedance (Zc) was taken as the average of the impedance amplitudes at harmonics with frequency values between 2 and 10 Hz, excluding amplitude values that were greater than two standard deviations above the mean amplitude. Wave Intensity analysis (WIA) was also conducted to calculate the total forward and backward wave intensities. In short, WIA is an alternative method to Fourier-based impedance to understanding vascular hemodynamics. In contrast to traditional Fourier techniques examining blood pressure and flow as the summation of a set of sinusoidal waves, instead, the waves are seen as a set of successive wavefronts [23]. WIA allows for the examination of flux in energy per unit area of a vascular segment due to pressure waves originating from the LV or returning from the periphery due to wave reflection. Hydraulic work or energy lost due to inefficiencies or mismatches between the LV and vascular system were also calculated. Finally, a sensitivity analysis was conducted where central pressure derived from the SphygmoCor rather than brachial pressure was used for patient-specific domain bounding criteria. This was done to assess the effect associated with using the generalized transfer function to convert brachial to central aortic pressure in AS patients.

### Statistical Analysis

Continuous data were presented as means with standard deviations and categorical variables were presented as counts and percentages. Absolute differences in vascular parameters from each impedance calculation method were compared using measured impedance and SBVI techniques. Bland–Altman plots were used for visualization and to assess bias and limits of agreement [9]. Given the lack of other methods for the non-invasive determination of vascular parameters for comparison of difference in methods, we future expressed the maximum 95% limit of agreement as a percentage of the standard deviation of the simulated metrics to better define the magnitude of the difference in the simulated method. Differences between measurements were calculated as non-invasively recorded minus simulated impedance. Pearson’s correlation coefficients for each set of metrics were also calculated. Point-by-point differences and the root mean squared error (RMSE) between central pressure waveforms derived from SBVI and recorded SphygmoCor central pressure waveforms were also calculated and compared. Finally, a sensitivity analysis was conducted to explore the relationship between input blood pressure measurement error and output vascular parameter error. Input blood pressure was varied by 10% in both the positive and negative direction for both systolic and diastolic values. Percent change from simulation vascular parameters at both upper and lower bounds were then compared.

## Results

### Cohort Characteristics

Data from a total of 111 patients undergoing TAVR who had sufficient echocardiographic and SphygmoCor data to complete both simulated and recorded vascular impedance measurements were used. The average age of the cohort was 73 years (SD 19), with 41% female, and 83% had hypertension at the time of baseline assessment. Overall hemodynamics were typical for patients with severe symptomatic AS. Group average AVA was 0.71 cm^2^ (SD 0.26), mean gradient 47 mmHg (SD 16), and a peak velocity of 425 cm/s (SD 79) (Table 1). From the initial generation of 500 million potential impedance values and resulting simulated central pressure waveforms, a mean of 25 waveforms was generated for each case.

**Table 1:** Pre-TAVR baseline demographic and hemodynamic variables

Characteristic	Baseline, *N* = 111^a^
Demographics	
Age (years)	74 (15)
Sex	
Male	66 (59%)
Female	45 (41%)
Body mass index	29.2 (6.7)
Hypertension	92 (83%)
Hemodynamics	
Systolic blood pressure (mmHg)	138 (21)
Diastolic blood pressure (mmHg)	75 (13)
Heart rate (bpm)	75 (12)
Mean gradient (mmHg)	47 (16)
Peak aortic velocity (cm/s)	425 (79)
Aortic value area (cm^2^)	0.71 (0.26)
Dimensionless index	1.37 (12.27)
Stroke volume (ml)	74 (27)
Ejection duration (s)	0.336 (0.032)
Acceleration time (s)	0.119 (0.022)
AVACT/ED ratio	0.36 (0.06)

^a^Mean (SD); *n* (%)

### Central Pressure Waveforms

A visual comparison between simulated and SphygmoCor-based central pressure waveforms showed good overall agreement (Online Appendix Fig. 7). After alignment of central waveforms, point-by-point differences across all patients averaged 4.01 mmHg (CI 3.9, 4.1 mmHg) with a small underestimation found with the simulation-based method. Root Mean Squared error averaged 4.81 mmHg (CI 4.7, 4.9 mmHg). Similarly, sensitivity analysis using the central pressure from the SphygmoCor for the patient-specific domain bounding decreased point-by-point waveform differences to 2.95 mmHg (CI 2.16, 4.34 mmHg), with an RMSE of 3.56 mmHg (CI 4.1, 4.3 mmHg).

### Vascular Parameters

**Fig. 2:**
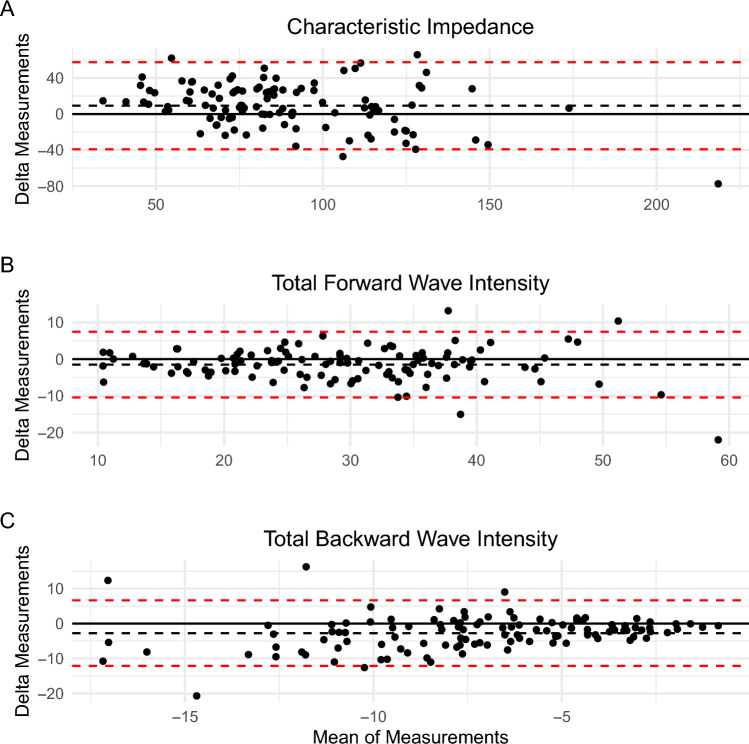
Bland–Altman plots showing characteristic impedance (dynes/cm^3^), total forward and backward wave intensity (W*m^−2^*s^−1^*1e4) values. The black dashed line indicates the overall mean value and dashed red lines the 95% limits of agreement. The delta of the measurements was taken as recorded minus simulated

**Fig. 3:**
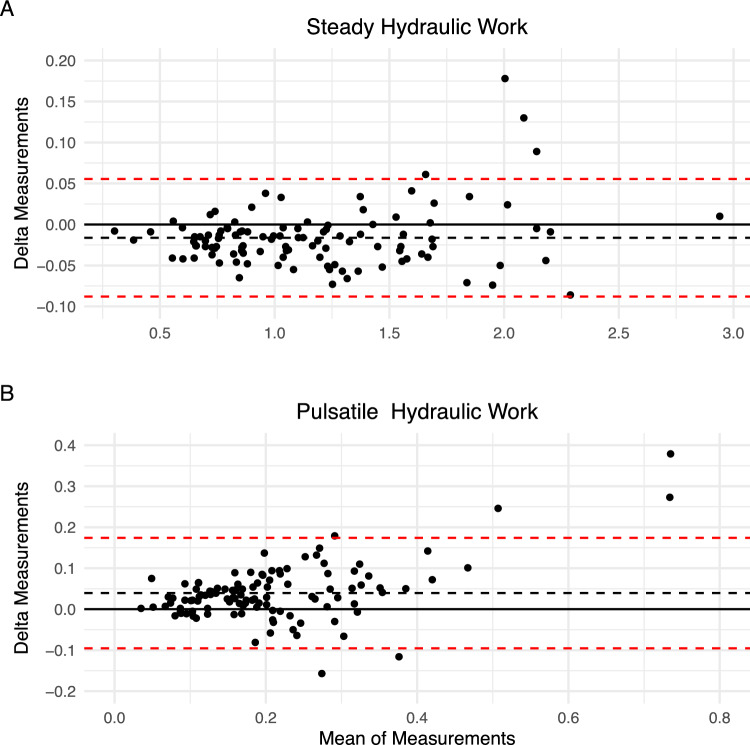
Bland–Altman plots steady and pulsatile hydraulic work (watts). The black dashed line indicates the overall mean value and dashed red lines the 95% limits of agreement. The delta of the measurements was taken as recorded minus simulated

Mean bias of the characteristic impedance (9.2 dynes/cm^3^) and steady hydraulic work (0.02 watts) indicated on average overestimation of simulation values compared to recorded values. On average total forward (− 1.5 Watts*m^−2^*s^−1^*1e4) and backward (− 2.7 Watts*m^−2^*s^−1^*1e4) wave intensities as well as pulsatile hydraulic work (− 0.04 watts) were underestimated relative to simulation-based values (Table 2). Visual inspection of Bland–Altman plots showed agreement between recorded and simulated measurements decreased as the measurement increased in magnitude (Figs. 2, 3). Correlation coefficients across metrics showed a high degree of correlation (*R*^2^ = 0.72 to 0.99) except for total backward wave intensity (*R*^2^ = 0.4) (Online Appendix Figs. 8, 9, 10, and 11). The magnitude as a percentage of the 95% limits of agreement was large, relative to the standard deviation of the recorded metric (Table 2), for several of parameters. As a percentage of the standard deviation, the larger limit of agreement was twice the standard deviation for characteristic impedance, 1.05 times the total forward wave intensity, 2.5 times the total backward wave intensity, 0.2 times the steady hydraulic work, and 1.0 times the pulsatile hydraulic work. Poor agreement was also seen in raw impedance amplitude values, except for the 0th harmonic which displayed a maximum limit of agreement of only 0.25 times the standard deviation. High correlation between recorded and simulated values were seen in all amplitude harmonics except for the 3rd harmonic. (Online Appendix Fig. 9 and Online Appendix Table 2).

**Table 2:** Comparison of vascular parameters calculated from non-invasively recorded and simulated methods for determination of vascular impedance

Metrics *N* = 111	Recorded (Mean ± SD)	Simulated (Mean ± SD)	Correlation coefficient	Mean bias	95% limits of agreement	Maximum limit of agreement as a percentage of SD
Frequency domain parameters						
Characteristic impedance (dynes/cm^3^)	84 (36)	93 (28)	0.72	9.2	− 39.2, 57.7	206
Total wave intensity analysis parameters						
Forward (Watts*m^−2^*s^−1^*1e4)	30 (11)	29 (10)	0.91	− 1.5	− 10.5, 7.4	105
Backward (Watts*m^−2^*s^−1^*1e4)	− 5.9 (3.8)	− 8.7 (4.8)	0.40	− 2.7	− 12.1, 6.7	252
Hydraulic work						
Steady (Watts)	1.16 (0.49)	1.17 (0.48)	0.99	0.02	− 0.06, 0.09	18.8
Pulsatile (Watts)	0.23 (0.14)	0.19 (0.10)	0.88	− 0.04	− 0.2, 0.1	200

Sensitivity analysis showed a 10% input error in blood pressure leading to a maximum of 69% output error relative to recorded values in backward wave intensity. All other vascular parameters showed no greater than a 30% output error. However, correlation coefficients and magnitude of mean bias were similar to that of simulation result without any artificial input error (Online Appendix Tables 3, 4, and 5).

## Discussion

We developed and validated a novel method of determining vascular input impedance, wave intensity, and hydraulic work using only echocardiographic data and standard clinical brachial blood pressure. The primary finding of this study was that simulation-based vascular impedance can provide metrics of vascular function similar to those measured directed using non-invasively inputs. Excellent agreement was seen in simulation and measured central pressure waveforms. With respect to impedance metrics, high correlation was found between recorded and simulated metrics for characteristic impedance, hydraulic work, and forward wave intensity metrics. However, the 95% limits of agreement were large for most of the parameters, and a small degree of bias was also associated with each metric. For prediction, correlation is more important than Bland–Altman agreement, however, in other contexts, it may not be valid to substitute the simulated for the actual metrics. The measurement of vascular impedance is not new but the technique itself has been sparsely used in the AS space. The majority of investigations have been limited to acute post-TAVR studies with longitudinal studies nonexistent. Due to the need for repeated pressure and flow measurements and the traditionally invasive nature to capture this data, the longitudinal progression of impedance in the general population has also been limited. With the adoption of echocardiography for flow measurements, larger-scale population-based studies of impedance became possible. For example, use of vascular impedance in the Framingham Heart Study allowed for the broad population-level understanding of ventricular–vascular coupling and potential risk factors for cardiovascular disease [7, 18, 19]. However, these studies have required long follow-up and specialized equipment. Measurement of impedance in AS patients has presented further challenges due to the blood flow jet produced by the narrowing AV. Narrowing of the valve creates a venturi effect causing a pressure drop near the valve along with an increase in jet velocity as is traditionally measured by continuous wave echocardiography. In the case of invasive assessment, this requires special attention to the catheter position for pressure and flow measurements [22, 28]. By further simplifying the data required for the calculation of metrics of vascular state, to those routinely known and collected by physicians, we can further lower barrier for the use of ventricular–vascular coupling metrics. Advances that allow for larger-scale and quick data collection can help translate these metrics from the research to clinical care domain.

Clinically, AS presents significant challenges as no pharmacological therapies are available, and treatment is limited to AVR as the disease progresses to its severe form. This paradigm of “watchful waiting” creates the need for novel metrics to better track disease course. Vascular afterload has been an aspect of disease progression that in the past has been either ignored or taken as simple systolic blood pressure. However, the dynamic time varying nature of the coupling of the LV and the AV requires techniques such as vascular impedance to be fully characterized. Our simulation-based method attempted to overcome measurement hurdles of the past and create a platform to allow for larger more detailed investigations of impedance in AS patients.

### Input Data Measurement Error

Given the simulation-based nature of our method, measurement error is limited to the input variables of the flow data and temporal distance between flow and pressure-based data capture. Prior work has shown that over the course of a standard clinical echocardiographic evaluation, systolic blood pressure can decrease as much as 12.4 mmHg [12]. This presents a logistical challenge, as without accurate measurements, errors will filter down within the analysis. To address this issue, we attempted to collect all pressure and flow data as close in time as possible, within 5 minutes of each other, to eliminate the chance of discordance and obtain as accurate measurements as possible.

An additional source of error is the generalized transfer function used in both the simulation method and SphygmoCor device, which we used for comparison of measured and simulation-based central pressure waveforms. Sensitivity analysis showed a small decrease in point-by-point waveform error when using central pressure only for patient-specific calibration. While this analysis does not remove the need for the generalized transfer function, by using the central aortic pressure measured non-invasively, it did not require an additional transformation within the simulation framework. While this did reduce the error associated with point-by-point differences, the benefit was relatively small given brachial pressure is nearly always measured clinically. Given the relatively small decrease of 0.53 mmHg in point-by-point and 0.61 mmHg by RMSE waveform difference, this may indicate that the use of the transfer function, while introducing some error, has limited effect on overall waveform morphology. Additionally, the sensitivity analysis of artificial variation in input blood pressure led to peak differences in recorded versus simulated metrics of 69% in backward wave intensity, with all other vascular parameters having errors below 30%. However, comparing these results to our primary analysis showed that all metrics had persevered correlation coefficients. We found similar results comparing mean bias between our original analysis and sensitivity analyses. While these differences may limit absolute value comparison, the use of these methods for research applications such as prediction modeling where correlation between variables can be more important than absolute values.

### Waveform Comparison

**Fig. 4:**
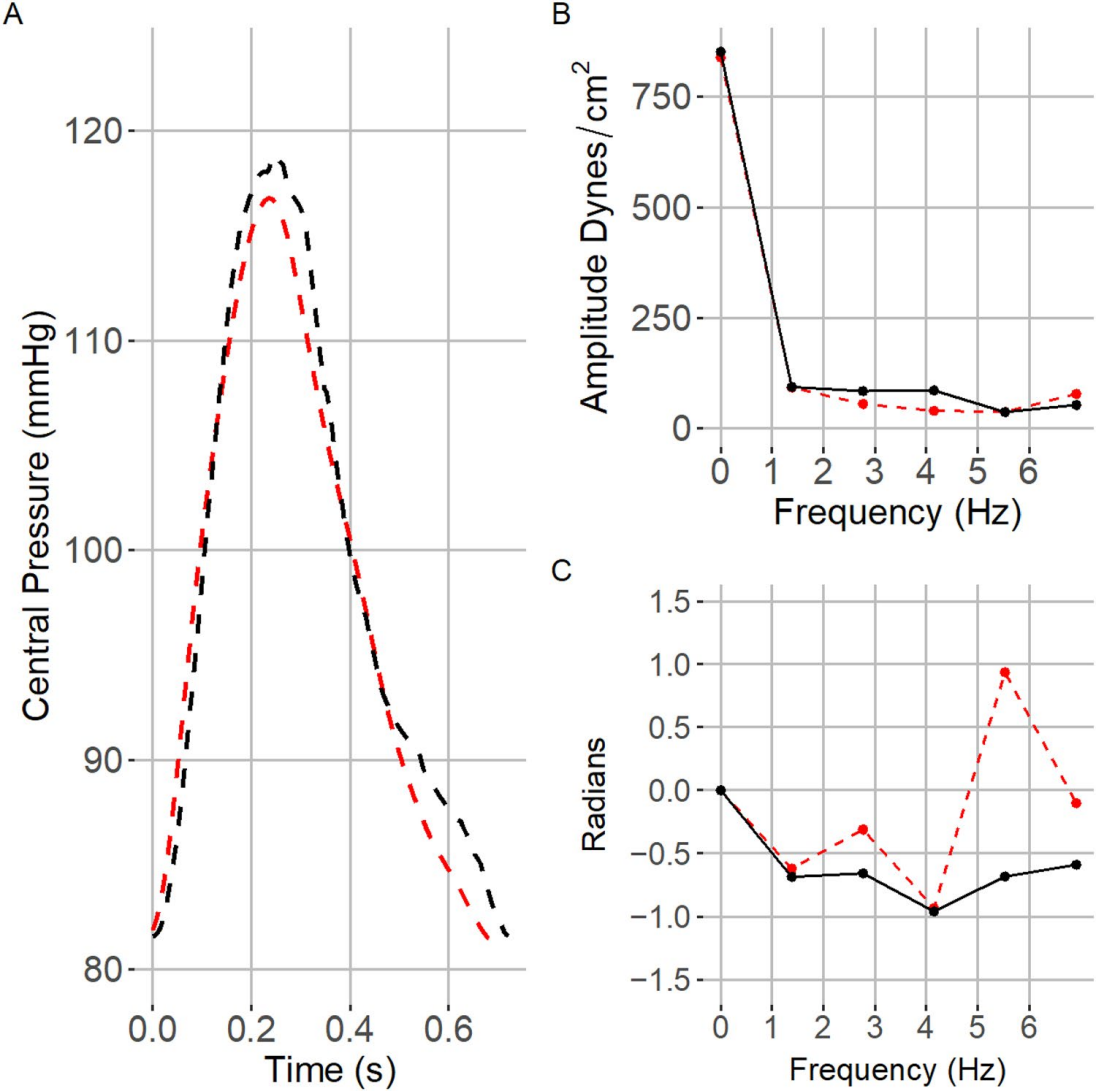
Example set of central pressure wave and impedance data is shown comparing measured and simulated values. **A** shows a comparison of non-invasively measured central pressure (dashed red), simulation-based central pressure waveforms that passed all domain bounding criteria (blue), and signal averaged median value (dashed black). **B** and **C** similarly show impedance amplitude and phase for the corresponding waveforms found in **A**

**Fig. 5:**
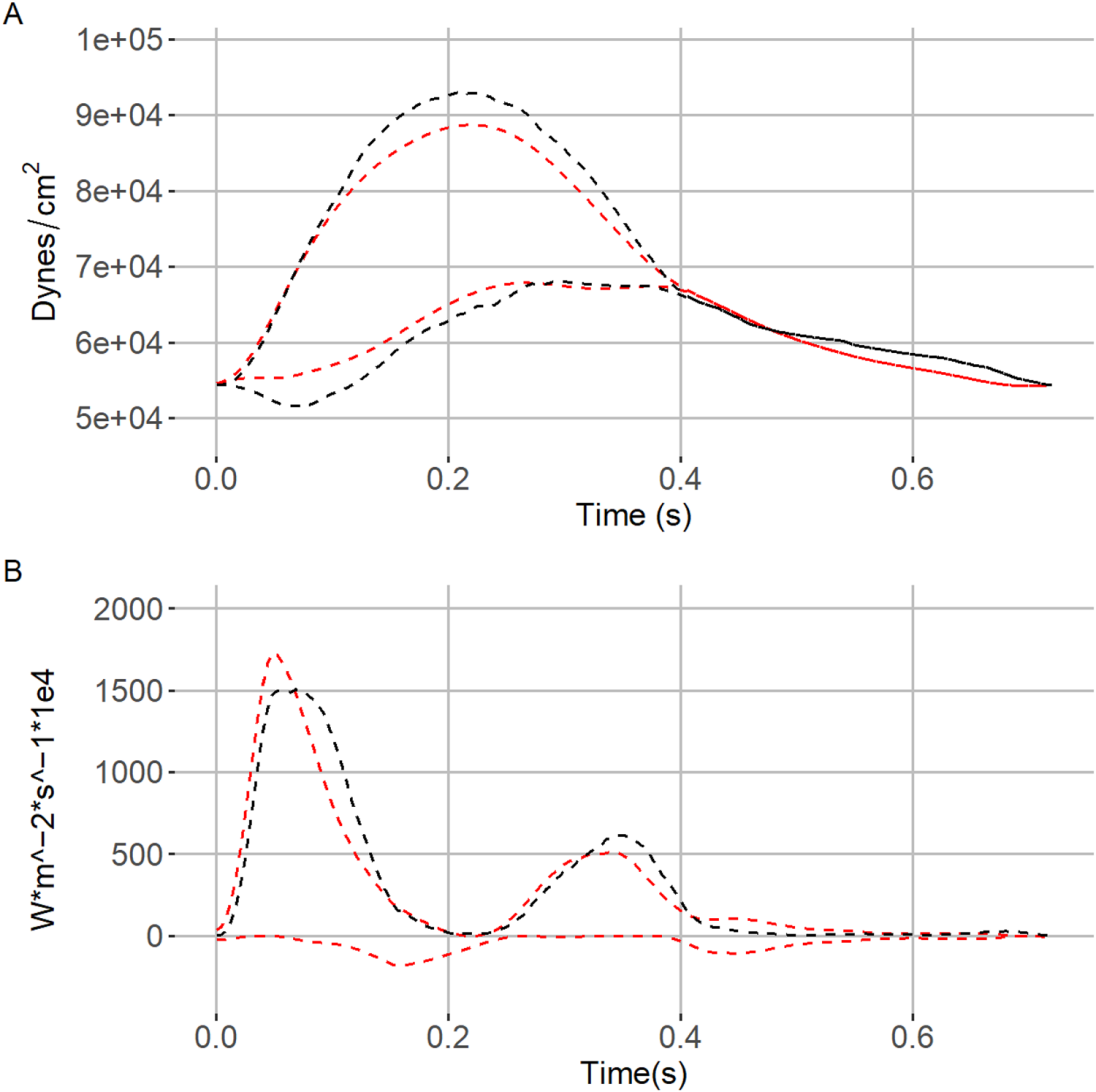
Example set of forward and backward pressure wave separation and wave intensity plots. **A** shows a comparison of non-invasively measured forward and backward separated central pressure (dashed red), simulation-based central forward and backward pressure waveforms that passed all domain bounding criteria (blue), and signal averaged median value (dashed black). **B** similarly show forward and backward wave intensity values for the corresponding waveforms found in **A**

Point-by-point comparisons of waveforms showed good agreement with only a 3.5 mmHg underestimation of central pressure relative to non-invasive measurement. As part of an invasive study of impedance in AS patients, Yotti et al. showed when measuring pressure using two different types of catheters, one of which was a high sensitivity pressure flow Combowire, differences up to 15 mmHg could be found [28]. However, morphological features of central pressure waveforms between devices showed little difference. A similar effect was seen with a visual inspection of our method in an example patient (Figs. 4, 5). This would suggest that while our simulation-based method does underestimate central pressure by a small amount this is similar to other invasive methods. Importantly, like non-invasive studies our simulation method maintains morphologic features as seen in the comparison of recorded and simulated central pressure waveforms across all subjects (Online Appendix Fig. 7). Furthermore, similar results have also been found when comparing the SphygmoCor device, used in this validation study, to invasive catheter-based measurements. In these studies, the mean differences in absolute value were found to be as large as 4.6 mmHg [25] but with a strong correlation between central systolic and pulse pressure [25].

### Impedance Spectrums

Due to the lack of scatter around the mean bias line, quantification of limits of agreement are not possible in the 0st through 3rd harmonics amplitude (Online Appendix Fig. 9). However, high degrees of correlation were seen in all but the 3rd harmonic. Given the known increase in noise with increasing harmonic, this finding is not surprising. Furthermore, most of the total impedance amplitude values are found in the first 2 or 3 harmonics with higher frequency values lumped together and taken as the characteristic impedance. Most of the impedance spectrum values found in this low-frequency range are the driving features of central pressure waveform morphology. One prior validation study that compared invasive to non-invasive techniques identified a tendency toward underestimation of impedance [14]. Despite this underestimation, high correlation between the invasive and non-invasive techniques were seen like in our study. This may be of particular importance when it comes to the clinical application of these techniques. While absolute differences in values were seen, high correlation among the simulation and recorded values may still allow for impedance-based variables to be used in prediction models. Finally, given the non-intuitive meaning of these variables, their clinical application is limited. Metrics derived from parts of this spectrum have been shown to be more clinically relevant such as wave intensity or hydraulic work [6, 21, 27].

### Characteristic Impedance

Limited prior literature exists on validation studies comparing methods of calculating impedance, and none in the AS population. Despite this, Kelly et al. [14] examined invasive to non-invasive calculation of impedance in patients undergoing left heart catheterization for coronary artery disease. While comparisons of impedance metrics across studies are challenging, given differences in how and which impedance metrics are calculated, we were able to compare characteristic impedance values. We found our own limits of agreement ranging from 57 to − 39 dynes/cm^3^, where Kelly et al. found approximately 75 to − 52 dynes/cm^5^. To directly compare the two studies, differences in how blood flow was measured need to be considered. In our study, we used linear blood flow from echocardiographic data, whereas Kelly at el. used similar echocardiographic data and then measured the valve area and multiplied the two to obtain volumetric flow. Converting one unit to the other would require a known aortic valve area for each patient, but if we take an assumed value for AS patients of 0.8 cm^2^, we obtain volumetric flow-based limits of agreement in our study of 71 to − 48 dynes/cm^5^, displaying similar results to those found in this prior study.

### Hydraulic Work and Total Wave Intensity

Simulated steady and hydraulic work showed high degrees of correlation to measured values, but poor agreement with respect to limits of agreement as a percentage of standard deviation. Furthermore, a tendency for values at the extremes were shown to have a greater degree of bias. In contrast, steady hydraulic work displayed excellent agreement by both correlation coefficient and limits of agreement as a percentage of standard deviation. This agreement was most likely due to the fact that the primary components in the calculation of steady hydraulic work are mean flow and mean pressure. Given the tight agreement between the measured and simulated central pressure waveforms and the use of the same flow data good agreement is not surprising.

Total wave intensity showed better agreement as measured by the correlation coefficient in the forward versus backward direction. Backward wave intensity relies upon accurate separation of the pressure wave into forward and backward components with the use of characteristic impedance. Similarly, in the time domain, this can be thought of as the acceleration time of the flow waveform, which is the point at which the pressure waveform is not yet affected by wave reflection effects. There is some debate as to whether calculation in the time or frequency domain is best for the determination of the characteristic impedance [4]. In our analysis, we chose to use the frequency domain-based calculation as the flow data for the measured and simulation-based methods were the same. Thus, any differences in any downstream calculations that required the use of characteristic impedance could be attributed to differences in the method used.

Correlation between characteristic impedance measured and recorded was similar despite the underestimation in the simulation-based data. This leads to the potential conclusion that while there was not good agreement between the total backward wave intensities any differences are most likely due to differences in how characteristic impedance was determined and ultimately the forward to backward separation point. However, in the time domain, the acceleration time differences are on the order of 20 ms indicating that trying to determine a difference this small may be more noise than signal. This is especially evident when, as with many echocardiographic-based measurements, the acceleration time is hand drawn and some inherent error is present in a person-to-person measurement. Taken together, this might suggest that while total backward wave intensity may be a high noise metric, that signal may be present provided that the same measurement technique is used.

### Limitations

As with any study, there are limitations to this validation. Some data have shown poor agreement between SphygmoCor and invasive measurements in AS patients, but these studies examined only absolute systolic and diastolic values and not waveform morphology [8]. This would also suggest that any differences in AS patients are not due to any inherent issues with the method but rather the use of the generalized transfer function in AS patients. However, in our sensitivity analysis using central rather than brachial pressure from the SphygmoCor, without the need for the generalized transfer function, we found similar agreement in the correlation of impedance metrics supporting the conclusion that the generalized transfer function does not add a significant amount of error. While input error on the order of 10% did induce output error of a greater degree, the agreement in the correlation of impedance metrics further supports that while accurate input data are important, for correlation-based tasks such as prediction, input error had minor effect. The development of an AS-specific generalized transfer function would further increase the accuracy of our simulation-based method. Simultaneous non-invasive capture of pressure and flow data is difficult. We attempted to reduce temporal mismatch as much as possible, however, differences between the measured pressure and flow data may have caused errors in downstream impedance measurements in both measured and simulated results.

Simulation-based vascular impedance can measure and calculate metrics of vascular function similarly to those measured non-invasively. The excellent agreement in central pressure waveforms and high correlation across a range of parameters positions this method well to calculate metrics for descriptive, associative, and predictive clinical research. Given the limited and standard clinical data required for our method, we believe it to be well positioned for use in large-scale retrospective studies to further explore both the acute and long-term effects of vascular changes over the course of AS.

## Supplementary Information

Below is the link to the electronic supplementary material.

Supplementary file1 (PDF 3277 kb)
