# Supplementary material for: Clinical Validation of Non-invasive Simulation-Based Determination of Vascular Impedance, Wave Intensity, and Hydraulic Work in Patients Undergoing Transcatheter Aortic Valve Replacement
Source: Ann Biomed Eng. 2024 Nov 19;53(2):536–46. doi: 10.1007/s10439-024-03635-5 (PMC11805843; doi:10.1007/s10439-024-03635-5)
Supplement: Supplementary file 1 — Supplementary file1 (PDF 3277 kb) [file 10439_2024_3635_MOESM1_ESM.pdf]

# Appendix - Clinical Validation of Non-Invasive Simulation Based Determination of Vascular Impedance in Patients Undergoing Transcatheter Aortic Valve Replacement

## Ejection Duration Optimization Algorithm

To obtain the patient flow velocity frequency spectrum, the patient time domain flow velocity waveform is first constructed. This is done in three steps using 5 required parameters: heart period, sample rate, maximum flow velocity, and input ejection duration and optional acceleration time. The units of the flow velocity waveform are cm/sec.

1. A parabola ($2^{nd}$-degree polynomial) is created to model the flow velocity waveform during the ejection period. The width of this parabola is given by the input ejection duration and the peak is given by the max flow velocity. If acceleration time is provided, the peak of the parabola is placed at the acceleration time. If acceleration time is not specified, the peak of the parabola is placed exactly in the center of the input ejection duration. The sample rate is used to determine time on the x-axis.
2. A vector of zeros is used to model the absence of flow from the end of the input ejection duration to the end of the heart period. The parabola is concatenated with this vector to create the complete flow velocity waveform.
3. The flow waveform is smoothed using $5^{th}$ order B-spline interpolation to avoid the Gibbs phenomenon. This smoothing results in a slightly widened ejection duration and shifts time of peak velocity.

While the heart period, sample rate, and maximum flow velocity are required program input variables, the input ejection duration, which is the ejection duration used to initially construct

the flow velocity waveform, is obtained from stroke volume (SV) as explained below. The input ejection duration is different from the ED (which is the ejection duration of the smoothed flow velocity waveform and therefore the actual patient ejection duration) due to the smoothing process described above – i.e. after the velocity waveform is smoothed, its ED is wider than the ejection duration that was used in its initial construction. Because of this, the processes described below are meant to obtain input ejection durations that will result in smoothed waveforms with ED values that match patient values.

Determination of input ejection duration:

1. Stroke Volume: The program will compute the input ejection duration from the given patient SV using an optimization process.
    a. A vector of 20 potential input ejection duration values is generated; it consists of values between 0.2 and 0.4 in increments of 0.01
    b. For each input ejection duration, a flow velocity waveform is constructed via the above method including smoothing.
    c. The velocity time integral of each generated flow velocity waveform is computed and the SV that is closest in value to the patient SV is determined.
    d. The input ejection duration that gave rise to this closest SV is then stored and used to construct the flow velocity waveform that will be used for the patient.

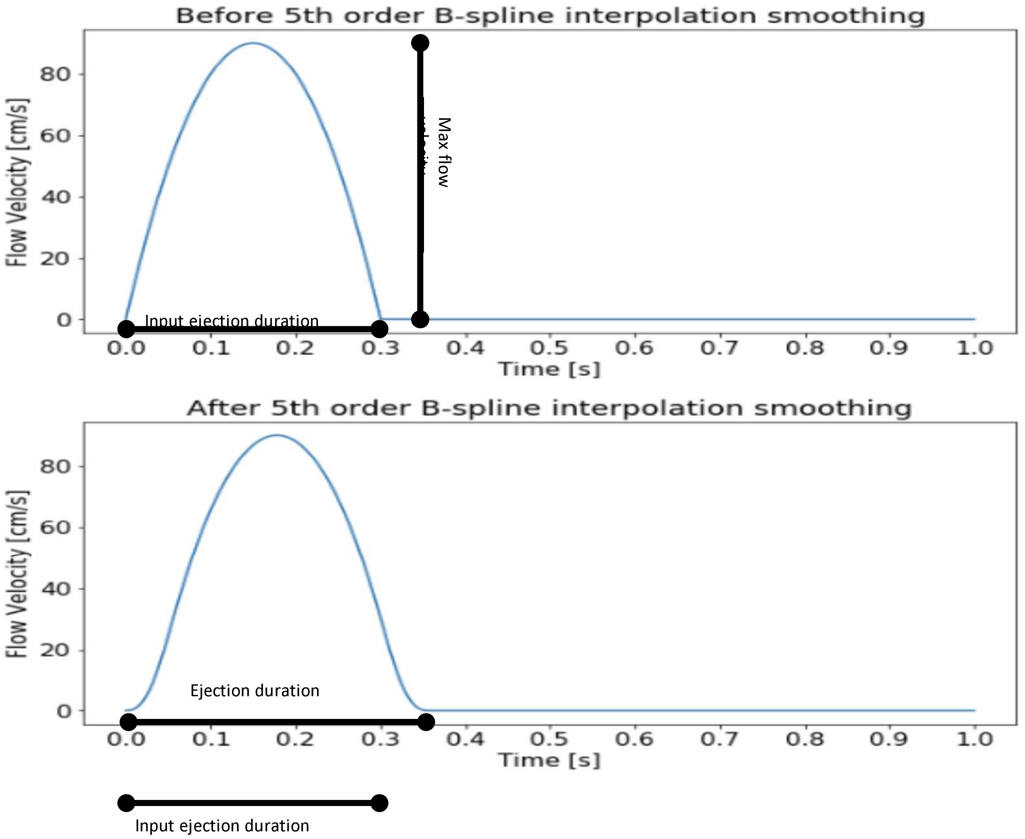


*Figure 6 Plot depicting the flow waveform before and after 5th order B-spline interpolation smoothing. Note that the ejection duration of the waveform after smoothing is somewhat increased compared to the input ejection duration prior to smoothing.*

## Calculation of Wave Intensity and Hydraulic Work

Calculation of all wave intensity values and hydraulic work was conducted using the following equations.

Hydraulic work [Watts]

a. Pulsatile: $\frac{1}{10^7} * \frac{1}{2} * [\sum_1^n (volumetric\ flow\ amplitude)_i{}^2 * (impedance\ amplitude)_i * \cos(impedance\ phase_i)]$ where volumetric flow amplitude is in cm^3/sec, impedance amplitude is in dyne*sec/cm^-5, and phase is in radians and wrapped.

b. Steady: $\frac{1}{10^7} * (mean\ volumetric\ flow) * (mean\ pressure)$ where mean volumetric flow is in cm^3/sec and mean pressure is in dynes/cm^2.

Wave separation & intensity analysis[1]

For separation of the forward and backward wave intensities the characteristic impedance calculated as the average of impedance amplitudes at harmonics with frequency values between 2-10 Hz, excluding amplitude values that are greater than two standard deviations above the mean amplitude. Impedance amplitudes are in dyne*sec/cm^3.

a. Forward pressure waveform component [dyne/cm^2]: 0.5*$(pressure\ waveform) + (characteristic\ impedance) * (flow\ waveform)$, where pressure is in dyne/cm^2, characteristic impedance is in dyne*sec/cm^3, and flow is in cm/sec

b. Backward pressure waveform component [dyne/cm^2]: 0.5* $(pressure\ waveform) - (characteristic\ impedance) * (flow\ waveform)$ , where pressure is in dyne/cm^2, characteristic impedance is in dyne*sec/cm^3, and flow is in cm/sec

c. Forward flow waveform component [cm/s]: 0.5* $\frac{forward\ pressure\ waveform\ component}{characteristic\ impedance}$, where forward pressure is in dyne/cm^2 and impedance is in dyne*sec/cm^3.

d. Backward flow waveform component [cm/s]: 0.5* $\frac{backward\ pressure\ waveform\ component}{characteristic\ impedance}$,

where backward pressure is in dyne/cm^2 and impedance is in dyne*sec/cm^3.

e. Forward wave intensity [W*m^−2*s^−2]:

$\frac{d}{dt}(forward\ pressure\ waveform\ component) *$

$\frac{d}{dt}(forward\ flow\ waveform\ component) * \frac{1}{1000}$, where forward pressure is in

dyne/cm^2 and forward flow is in cm/sec.

f. Backward wave intensity [W*m^−2*s^−2]:

$\frac{d}{dt}(backward\ pressure\ waveform\ component) *$

$\frac{d}{dt}(backward\ flow\ waveform\ component) * \frac{1}{1000}$, where backward pressure is in

dyne/cm^2 and backward flow is in cm/sec.

## Tables and Figure

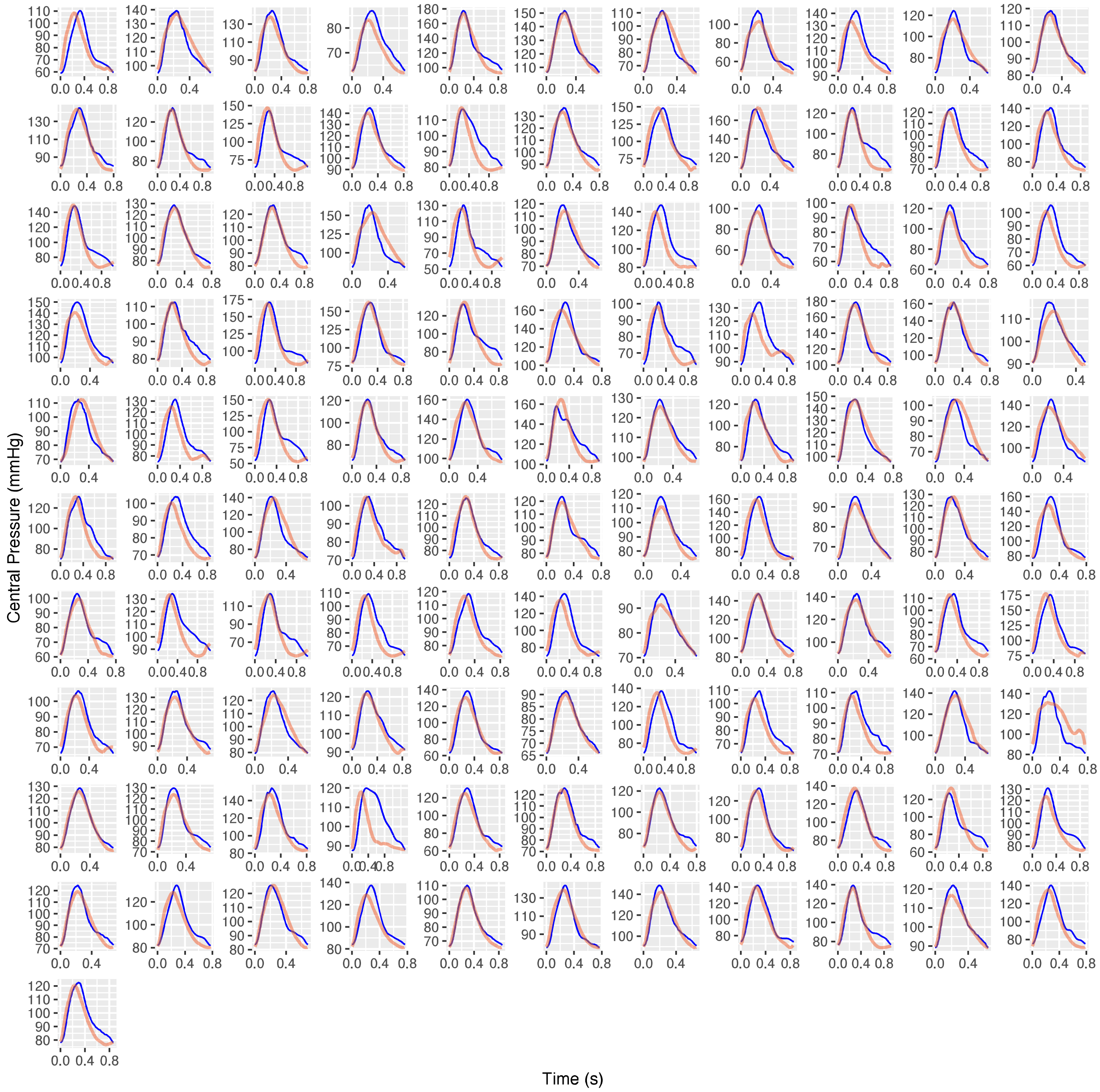


*Figure 7 Full set comparison of non-invasive central pressure measured via the SphygmoCor XCEL device (Red) and aortic central pressure generated from simulated data (Blue). Overall waveform morphology concordance between measured and simulated data was good as measured by point-by-point differences. A tendency toward underestimation of aortic central pressure from simulated results. Point by Point difference across all patients averaged 4.01 mmhg (CI: 3.9, 4.1 mmHg) with a Root Mean Squared Error of 4.81 mmHg (CI: 4.7, 4.9 mmHg)*

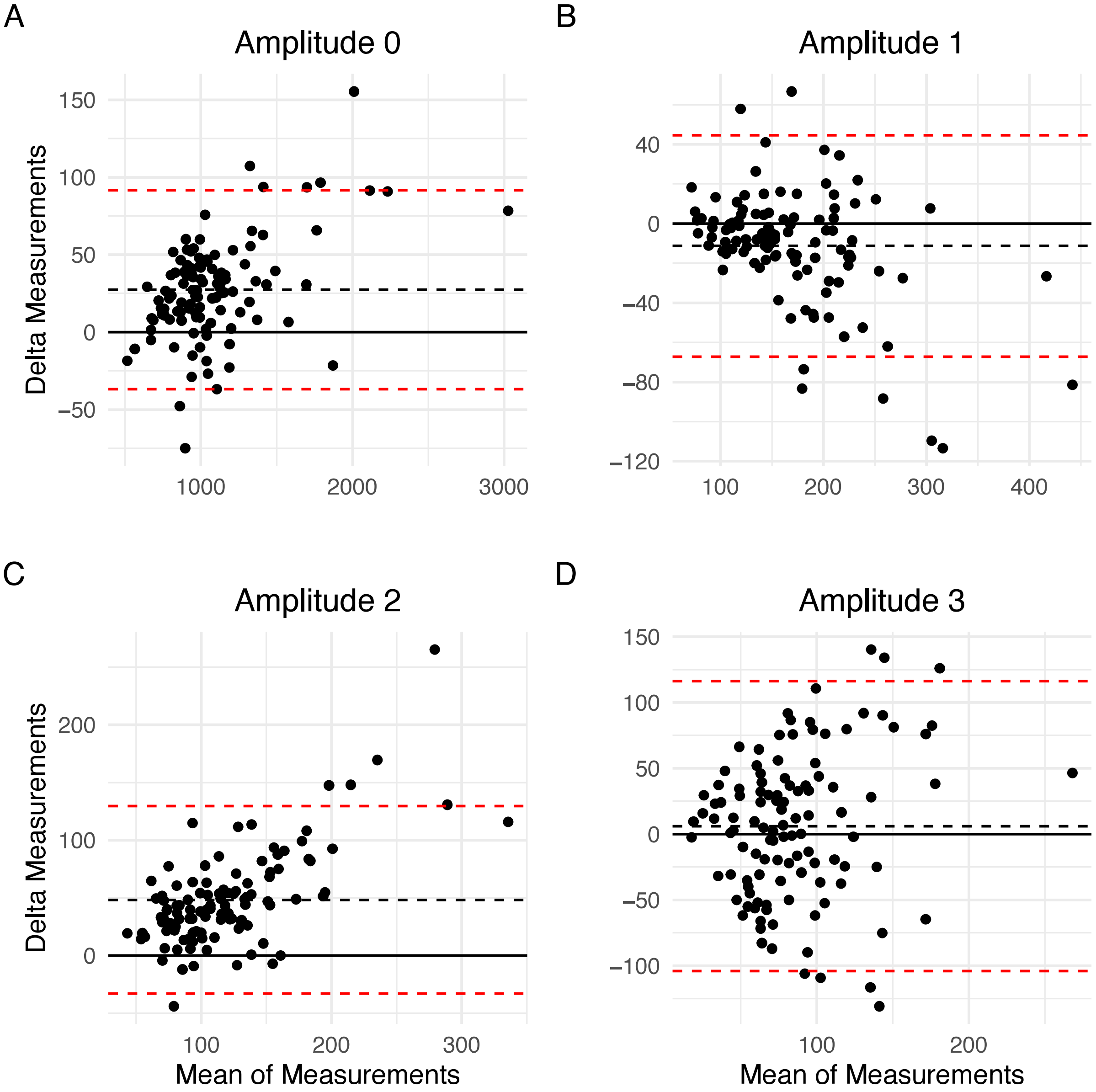


*Figure 8 Bland-Altman plots showing the mean of the measurements on the x-axis and differences in measurements on the y-axis. Raw harmonic amplitude values from the 0$^{th}$ to 3$^{rd}$ harmonic are shown. Units for all amplitude values are dynes/cm$^3$. The black dashed line indicates the overall mean value and dashed red lines the 95% limits of agreement. Due to*

*spread of values at higher values and non-random scatter around mean bias line limits of agreement cannot be used to fully assess agreement.*

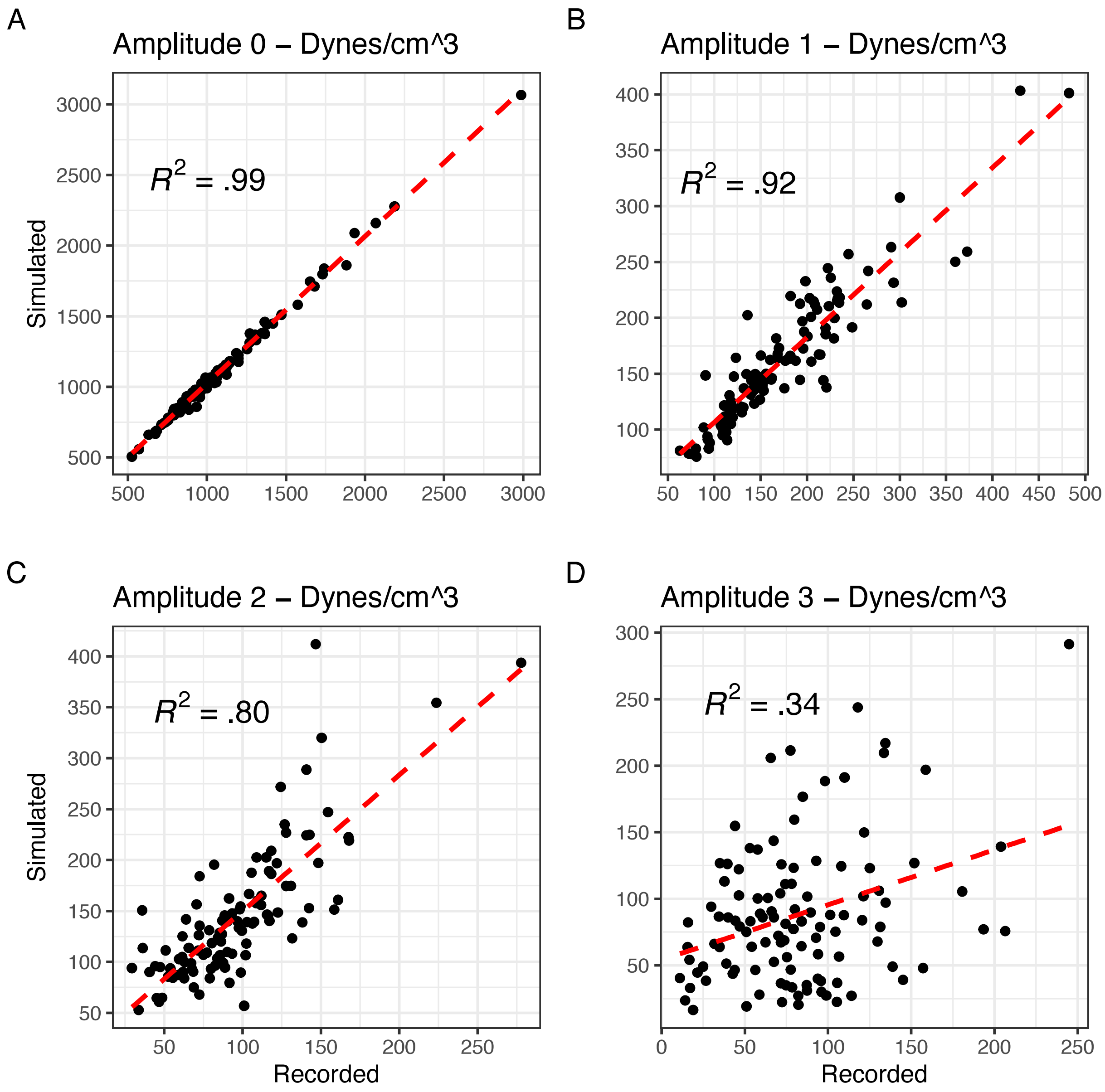


*Figure 9 Scatter plots comparing recorded and simulated impedance amplitude values. Red dashed line represents the best fit line.*

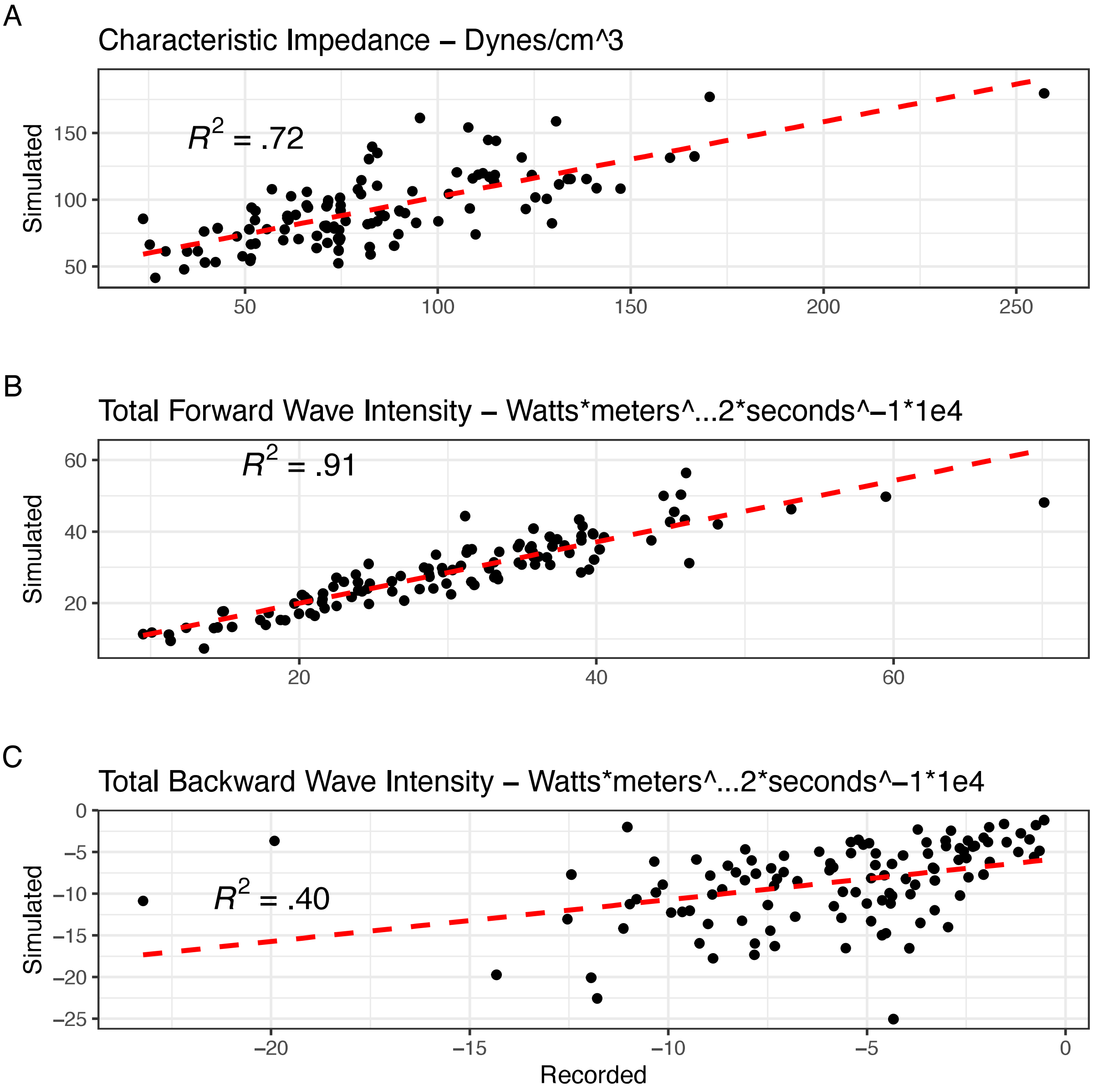


*Figure 10 Scatter plot comparing recorded and simulated vascular parameters of characteristic impedance, and total forward and backward wave intensities. Red dashed line represents the best fit line.*

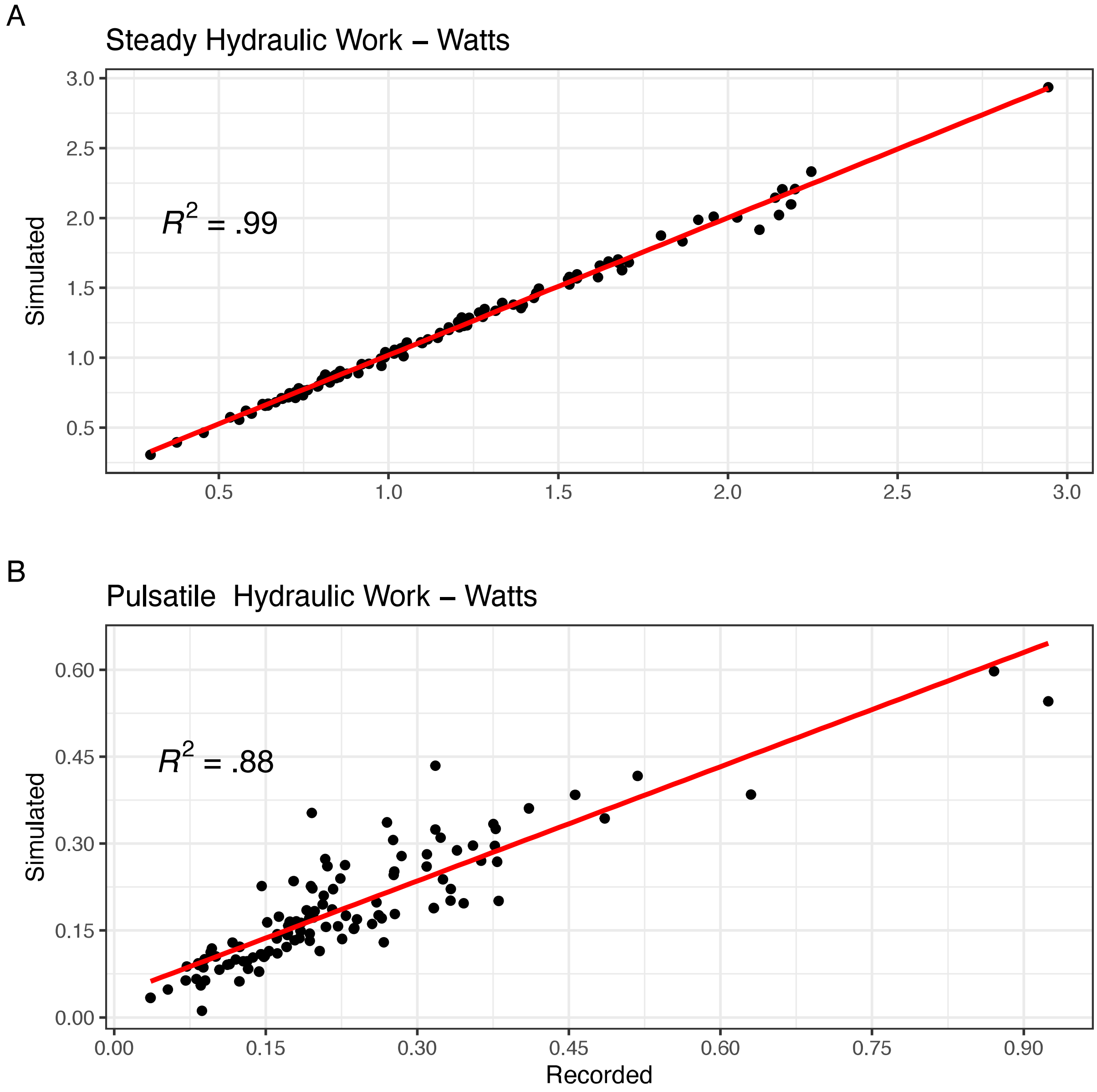


*Figure 11 Scatter plot comparing recorded and simulated steady and pulsatile hydraulic work. Red dashed line represents the best fit line.*

*Table 1 Comparison of frequency domain based vascular parameters calculated from non-invasive and simulated methods for determination of vascular impedance.*

| *Metrics N = 111* | *Recorded (Mean±SD)* | *Simulated (Mean±SD)* | *Correlation Coefficient* | *Mean Bias* | *95% Limits of Agreement* | *Maximum Limit of Agreement as a Percentage of SD* |
|---|---|---|---|---|---|---|
| <u>*Frequency Domain Parameters*</u> | | | | | | |
| *$0^{th}$ Amplitude Harmonic (dynes/cm$^3$)* | 1073 (352) | 1100 (368) | 0.99 | 27.4 | 91.6, -36.9 | 25 |
| *$1^{st}$ Amplitude Harmonic (dynes/cm$^3$)* | 174 (71) | 163 (59) | 0.92 | -11.3 | 44.7, -67.2 | 113 |
| *$2^{nd}$ Amplitude Harmonic (dynes/cm$^3$)* | 95 (39) | 143 (65) | 0.80 | 48.2 | 129.5, -33.0 | 199 |
| *$3^{rd}$ Amplitude Harmonic (dynes/cm$^3$)* | 82 (39) | 88 (53) | 0.34 | 6.02 | 116.2, -104.2 | 219 |

*Table 2. Summary of vascular function metrics from sensitivity analysis using central instead of brachial blood pressure*

| *Metrics N = 102* | *Recorded (Mean±SD)* | *Simulated (Mean±SD)* | *Correlation Coefficient* | *Mean Bias* | *95% Limits of Agreement* | *Maximum Limit of Agreement as a Percentage of SD* |
|---|---|---|---|---|---|---|
| | | *Frequency Domain Parameters* | | | | |
| *$0^{th}$ Amplitude Harmonic (dynes/cm$^3$)* | 1056 (310) | 1061 (320) | 0.99 | 5.7 | -50.8, 62.3 | 19.4 |
| *$1^{st}$ Amplitude Harmonic (dynes/cm$^3$)* | 168 (69) | 147 (58) | 0.97 | -22.2 | -56.9, 12.6 | 98 |
| *$2^{nd}$ Amplitude Harmonic (dynes/cm$^3$)* | 93 (39) | 135 (63) | 0.83 | 40.8 | -29.3, 110.9 | 176 |
| *Characteristic Impedance (dynes/cm$^3$)* | 82 (45) | 88 (27) | 0.73 | 4.6 | -44.6, 52.8 | 196 |
| | | *Total Wave Intensity Analysis Parameters* | | | | |
| *Forward (W*m$^{-2}$*s$^{-1}$*1e4)* | 29 (10) | 26 (10) | 0.95 | -3.3 | -9.1, 3.6 | 91 |
| *Backward (W*m$^{-2}$*s$^{-1}$*1e4)* | -5.5 (3.3) | -7.7 (4.1) | 0.51 | -2.2 | -9.6, 5.4 | 234 |
| | | *Hydraulic work* | | | | |
| *Steady (Watts)* | 1.16 (0.49) | 1.15 (0.48) | 0.99 | -0.01 | -0.08, 0.06 | 16 |
| *Pulsatile (Watts)* | 0.22 (0.12) | 0.17 (0.10) | 0.91 | -0.05 | -0.15, 0.6 | 6 |

# Sensitivity Analysis – Variation of Input Blood Pressure

## Lower Bound

*Table 3 Lower Bound of Input Error Sensitivity Analysis*

| *Metrics N = 44* | *Recorded (Mean±SD)* | *Simulated Lower Bound (Mean±SD)* | *Correlation Coefficient* | *Mean Bias* | *95% Limits of Agreement* | *Maximum Limit of Agreement as a Percentage of SD* |
|---|---|---|---|---|---|---|
| *Frequency Domain Parameters* | | | | | | |
| *Characteristic Impedance (dynes/cm$^3$)* | 99 (49) | 96 (231) | 0.82 | -2.6 | -60.4, 55.2 | 26 |
| *Total Wave Intensity Analysis Parameters* | | | | | | |
| *Forward (Watts*meters$^{-2}$*seconds$^{-1}$*1e4)* | 34 (11) | 28 (10) | 0.96 | -5.5 | -12.5, 1.5 | 125 |
| *Backward (Watts*meters$^{-2}$*seconds$^{-1}$*1e4)* | -5.2 (3.1) | -6.8 (2.8) | 0.30 | -1.7 | -8.5, 5.2 | 304 |
| *Hydraulic work* | | | | | | |
| *Steady (Watts)* | 1.07 (0.50) | 1.0 (0.46) | 0.99 | -0.08 | -0.17, 0.02 | 4.3 |
| *Pulsatile (Watts)* | 0.24 (0.15) | 0.18 (0.11) | 0.97 | -0.07 | -0.17, 0.04 | 36 |

## Upper Bound

*Table 4 Upper Bound of Input Error Sensitivity Analysis*

| *Metrics N = 43* | *Recorded (Mean±SD)* | *Simulated Upper Bound (Mean±SD)* | *Correlation Coefficient* | *Mean Bias* | *95% Limits of Agreement* | *Maximum Limit of Agreement as a Percentage of SD* |
|---|---|---|---|---|---|---|
| *Frequency Domain Parameters* | | | | | | |
| *Characteristic Impedance (dynes/cm$^3$)* | 96 (49) | 110 (33) | 0.8 | 14.1 | -45.2, 73.4 | 222 |
| *Total Wave Intensity Analysis Parameters* | | | | | | |
| *Forward (Watts*meters$^{-2}$*seconds$^{-1}$*1e4)* | 34 (12) | 35 (12) | 0.94 | 1.1 | -6.5, 8.7 | 72.5 |
| *Backward (Watts*meters$^{-2}$*seconds$^{-1}$*1e4)* | -5.2 (3.13) | -8.8 (4.7) | 0.34 | -3.6 | -12.9, 5.6 | 274 |
| *Hydraulic work* | | | | | | |
| *Steady (Watts)* | 1.1 (0.49) | 1.2 (0.55) | 0.99 | 0.14 | -0.0006, 0.28 | 50.9 |
| *Pulsatile (Watts)* | 0.23 (0.12) | 0.21 (0.12) | 0.94 | -0.02 | -0.1, 0.06 | 50 |

*Table 5 sensitivity analysis comparison between recorded and simulated vascular metrics at both the upper and lower bound of introduced input error.*

| Parameter | % Change (Upper, Lower bound of variation) |
|---|---|
| Characteristic Impedance | 3.03 %, -14.6 % |
| Forward | 17.7%, -2.9% |
| Backward | -30.8%, -69.2% |
| Steady | 6.5%, -9.1% |
| Pulsatile | 25%, 8.7% |

## Reference


1. Westerhof, N., P. Segers, and B. E. Westerhof. Wave separation, wave intensity, the reservoir-wave concept, and the instantaneous wave-free ratio: Presumptions and principles. *Hypertension* 66:93–98, 2015.
